# Few rights, great threats, endless struggles: setbacks and resistance in the last decade of fights for legal abortion in Brazil

**DOI:** 10.1080/26410397.2025.2555050

**Published:** 2025-09-03

**Authors:** Nathália Machado Cardoso, Mariana Pércia Namé de Souza Franco, Luiza Magalhães Cadioli, Ana Flávia Pires Lucas d'Oliveira, Elisabeth Meloni Vieira

**Affiliations:** a General Practitioner, Doctoral Student in Collective Health, Department of Preventive Medicine, Faculty of Medicine, University of São Paulo, São Paulo, Brazil. *Correspondence*: nathalia.mc@usp.br; b Gynaecologist and Obstetrician, Master's student, School of Public Health at the University of São Paulo, São Paulo, Brazil; c General Practitioner, Master's student, School of Public Health, University of São Paulo, São Paulo, Brazil; d Professor, Department of Preventive Medicine, University of São Paulo School of Medicine, São Paulo, Brazil; e Associate Professor, Department of Health, Cycles of Life and Society, School of Public Health, Universidade de São Paulo, São Paulo, Brazil

**Keywords:** legal abortion, reproductive rights, gender ideology, gender and politics, political conservatism, reproductive justice

## Abstract

This commentary analyses the state of legal abortion in Brazil over the past decade, contextualising the increasing restrictions and political disputes surrounding the issue within broader anti-gender offensives. While Brazilian law permits abortion only in limited cases – rape, risk to the pregnant person's life, and anencephaly – access to these rights has been consistently undermined, particularly amid the strengthening of far-right political forces. We explore how moral arguments and conservative discourses – often framed through the notion of “gender ideology” – have been mobilised to roll back sexual and reproductive rights, resulting in significant institutional and legislative setbacks, including attempts to criminalise legal abortion practices. In contrast, the commentary highlights forms of resistance led by feminist movements, progressive lawmakers, and the judiciary, such as mass protests, strategic litigation, and efforts to socially decriminalise abortion. Furthermore, it addresses the role of strategic ignorance in perpetuating state inaction and the importance of academic research in illuminating these dynamics and resisting the erosion of rights. The struggle for reproductive justice in Brazil is ongoing, marked by both persistent threats and collective resistance.

## Résumé

Ce commentaire analyse la situation de l’avortement légal au Brésil au cours de la dernière décennie, en contextualisant les restrictions croissantes et les différends politiques entourant cette question dans le cadre d’offensives anti-genre plus larges. Si la loi brésilienne n’autorise l’avortement que dans des cas limités – viol, risque pour la vie de la personne enceinte et anencéphalie – l’accès à ces droits a été systématiquement sapé, en particulier dans un contexte de renforcement des forces politiques d’extrême droite. Nous étudions la manière dont les arguments moraux et les discours conservateurs, souvent formulés à travers la notion d’« idéologie du genre », ont été mobilisés pour faire reculer les droits sexuels et reproductifs, entraînant d’importants revers institutionnels et législatifs, notamment des tentatives de pénalisation des pratiques d’avortement légal. Par opposition, le commentaire met en lumière les formes de résistance dirigées par des mouvements féministes, des législateurs progressistes et le pouvoir judiciaire, comme les manifestations de masse, les litiges stratégiques et les efforts visant à dépénaliser socialement l’avortement. De plus, il aborde le rôle de l’ignorance stratégique dans la perpétuation de l’inaction de l’État et l’importance de la recherche universitaire pour éclairer ces dynamiques et résister à l’érosion des droits. La lutte pour la justice reproductive au Brésil est en cours, marquée à la fois par des menaces persistantes et par une résistance collective.

## Resumen

Este comentario analiza el estado del aborto legal en Brasil en la última década, y contextualiza las crecientes restricciones y disputas políticas en torno al asunto dentro de ofensivas antigénero más amplias. Si bien la ley brasileña permite el aborto solo en casos limitados — violación, riesgo para la vida de la persona embarazada y anencefalia— el acceso a estos derechos se ha visto socavado una y otra vez, en particular en medio del fortalecimiento de las fuerzas políticas de extrema derecha. Exploramos cómo los argumentos morales y discursos conservadores, a menudo planteados por medio de la noción de “ideología de género”, se han movilizado para revertir los derechos sexuales y reproductivos, lo cual ha causado considerables reveses institucionales y legislativos, tales como intentos por penalizar las prácticas de aborto legal. En cambio, el comentario destaca formas de resistencia liderada por movimientos feministas, legisladores progresistas y la judicatura, tales como protestas masivas, litigio estratégico y esfuerzos por despenalizar el aborto socialmente. Además, aborda el papel de la ignorancia estratégica en perpetuar la inacción estatal y la importancia de la investigación académica en iluminar estas dinámicas y resistir la erosión de los derechos. La lucha por la justicia reproductiva en Brasil es continua y está marcada tanto por amenazas persistentes como por resistencia colectiva.

Brazilian abortion law is among the most restrictive in the world,^1^ allowing the procedure only in cases of sexual violence, risk to the pregnant person's life, or anencephaly,^2,3^ with the subject often treated discreetly by society. Nevertheless, the obstacles to accessing even these limited rights have become increasingly central to public life, especially over the past decade. During this period, gender and sexuality have become key battlegrounds, shaping political agendas, polarising public discourse, and triggering both moral backlash and organised resistance.^4,5^

After the end of the military dictatorship in the 1980s, democratically elected presidents committed themselves to the defence of human rights and guaranteed Brazil's compliance with fundamental international commitments.^6,7^ These include those established at the United Nations conferences held in the 1990s, dedicated to the protection and assurance of reproductive and sexual rights.^8^

Public education and health policies have been expanded and universalised,^9^ and much progress has been achieved in consolidating social minoritised majorities’^10^ rights, including specific policies to protect black, poor, indigenous, LGBTQIAP+, homeless and landless rural workers.^9^ Comprehensive women's health programmes have been organised to overcome the exclusive focus on maternal and child health, with interventions to combat gender-based violence, obstetric and institutional abuse, expand the supply of contraceptives and prevent sexually transmitted infections,^11,12^ all incorporating the demands of feminist movements.^6^

However, consolidating a political agenda in favour of women and other marginalised groups between 2003 and 2014 did not come without attacks or threats of backlash. This commentary seeks to situate the setbacks against the abortion agenda in Brazil within a broader panorama of conservative reactions, in which the so-called “gender ideology” plays a relevant role. This accusatory category^13^ extends from macro-politics to everyday life and has direct implications for the guarantee of sexual and reproductive rights. We highlight how these dynamics affect girls’, women's and pregnant people's lives, and explore the institutional and social movement responses to these setbacks. The analysis covers the period between the parliamentary coup of 2016, through the Temer and Bolsonaro administrations, to Luiz Inácio Lula da Silva’s third term in Presidency.

## Undoing two decades of progress: the moral backlash against gender and sexual rights and the resurgence of the far right in Brazil

The democratic progress experienced in Latin America between the late 1980s and the first decade of the 2000s overshadowed the way reactionary and ultra-conservative forces, both secular and religious, were undergoing an intense process of reconfiguration and strengthening in Europe and the United States.^5^ This transformation in the behaviour and strategies of the political right meant a change in focus: previously only reactionary and defender of order, it became an engine of political mobilisation, contesting values and common sense, and confronting feminism and the LGBTQIAP+ movement on issues such as abortion, sexual identity and family models.^5^

In 1997, the term “gender ideology” was used for the first time in the ultra-conservative camp, marking the beginning of systematic intellectual work to consolidate its accusatory character in the transnational arena made possible by the transition to globalisation and intensified communication.^5^

In Brazil, as in other Latin American countries, the anti-gender offensives have overlapped with the old, generally Catholic, infrastructures of opposition to sexual and reproductive rights, especially the right to abortion.^5^ For Sônia Correa,^5^ they have multiple facets and act in several ways at once, which makes it difficult to find a simple explanation or a single cause for their actions. Moreover, they are not just a backlash or predominantly religious, but more complex phenomena, driven by a combination of different social and ideological forces.^5^

The link between gender and Marxism meant that the offensive achieved the goal of occupying the Brazilian State, combining the unleashing of moral panics^4^ with a strong sense of anti-communism and rejection of egalitarianism, factors that contributed to the rise of the far right to power in 2018.^5^

After the parliamentary coup that ousted the first and only elected female president, Dilma Rousseff, in 2016, conservative proposals to roll back a wide range of civil, political and social rights were strengthened at federal, state and municipal levels.^9^ There has been an institutional hollowing out with the extinction of women's secretariats and ministries, and resources for women’s policies have been cut by more than half.^13^ There has also been a deterioration in equity policies, which intensified after the extreme right came to power, with Bolsonaro in the Presidency of the Republic and an evangelical pastor in the Ministry of Women, Family and Human Rights, created after the dismantling of the Women’s Ministry.^4,14^

Even before the coup, conservative and religious politicians were trying to approve laws that would restrict access to legal abortion. One example was the Constitutional Amendment Bill 29/2015,^15^ which proposed to include the “right to life from the moment of conception”, criminalise the use of the emergency pill and any voluntary interruption of pregnancy, and prevent in vitro fertilisation procedures and stem cell research. The proposal, submitted by a senator and an evangelical pastor, was never voted on in plenary and ended up being shelved in December 2022.

Equating the embryo to a citizen is the basis for a wide range of projects aimed at violating the reproductive rights of women, girls, and pregnant people in Brazil. Examples of this are the bills that propose to classify abortion as a heinous crime, to criminalise professionals who carry out the procedure, to prevent rape victims from accessing it,^16,17^ and even to deny access to contraceptive methods. All those measures have been strengthened after the election of a self-proclaimed “pro-life” government.

An example of this was the annulment of the technical note issued in June 2020,^18^ which recommended the continuity of health services to guarantee access to emergency contraceptive methods and abortion permitted by law during the COVID-19 pandemic, in accordance with international standards.^19^ The suspension of the note, accompanied by the dismissal of its authors from their posts, became the first milestone in a series of events that demonstrate the State's neglect of women's health, especially those who rely exclusively on the public system.^20^

Two months later, in a scenario where several countries were proposing strategies to reduce inequalities in the midst of the crisis imposed by the pandemic, the Ministry of Health (MoH) made it compulsory to report cases of pregnant women seeking abortion after sexual violence to the police within 24 hours,^21^ violating the right to confidentiality and threatening the right to health. Other measures have included the imposition of intimidating procedures such as “visualisation of the foetus or embryo by ultrasound” and listening to foetal heartbeats, which have been interpreted as a crime of torture.^20^

In 2022, the Primary Health Care Secretariat issued a note stating that abortions should not be performed after 21 weeks due to fetal viability, thus restricting the right to legal abortion in these cases, in an illegal position in relation to the Penal Code, which has no gestational age limit for abortion.^2^

Six months before the end of Bolsonaro's government, the MoH published the manual “Technical attention for prevention, evaluation and conduct in cases of abortion”,^22^ considered unscrupulous by the Brazilian Association of Collective Health.^23^ The document, which replaced the previous “Humanised abortion care”,^24^ aimed to make legal abortion in Brazil as difficult as possible, disqualifying legal permissions, defending the protection of life from conception and creating barriers to access, such as the prohibition of abortion by telemedicine. Contrary to the recommendations of the World Health Organization (WHO), it promoted the violation of professional secrecy and did not recognise maternal death due to abortion as a public health issue, cynically maintaining concepts such as autonomy and acceptance.^23,25^

In 2023, Lula was re-elected, but the strengthening of the right and the polarisation around moral issues made the issue sensitive for the Executive.^14^ This phenomenon reflects a broader pattern in Latin America, where many governments have chosen to “turn a blind eye” to the issue, fearing the high political costs of defending reproductive rights or implementing reforms and shirking their responsibilities.^26^ Since the transition of government, the predominant attitude has been to avoid the issue so as not to increase polarisation or mobilise conservative bases.

For Sônia Correa,^5^ the ultra-conservatism of Brazilian society, intensified with the gender wars, can be explained by the intersection of three long-term systemic trends. The first is the trajectory of re-democratisation, characterised by deficits in democratic regimes and the persistence of authoritarianism and violence. The second is the influence of neoliberalism, which has generated inequality, social insecurity and the erosion of the political sphere. Finally, the third trend is the growing politicisation of religious ultra-conservatism. This is evident in the composition of the current National Congress, which is elected by the Brazilian people and is increasingly aligned with far-right and neo-conservative ideologies. Currently, around 30% of parliamentarians belong to the Parliamentary Front against Abortion and in Defense of Life,^27,28^ a group with strong alliances with the pro-gun and agribusiness sectors and opposed to the feminist agenda, especially on issues related to bodily autonomy and sexual and reproductive health.^4,14,29^

## Attacks, effects and resistance

An emblematic episode in the ongoing power game occurred in early 2024, with the publication of a note by the MoH. In accordance with the Penal Code, the note repealed the gestational age limits for the legal interruption of pregnancy, cancelling both the manual of June 2022 and the technical note associated with it. The measure provoked a reaction from the evangelical caucus in the National Congress and the conservative media, which led the MoH to suspend the text in less than 24 hours.^30–32^

A few months later, the Federal Council of Medicine (CFM), with its current administration aligned with reactionary views and the fight against women's rights, issued a resolution prohibiting the use of asystole in abortions after 22 weeks of gestation.^33^ which, in practice, criminalised abortion after this gestational age and prevented appropriate medical behaviour in these cases. This decision was suspended by the Supreme Federal Court (STF) on the grounds that the CFM had abused its regulatory power and distanced itself from internationally recommended scientific practices. Administrative and disciplinary proceedings brought against professionals as a result of the resolution were suspended. But the response was not long in coming, in the form of two new attacks.^34^

The first came from the President of the Chamber of Deputies, Arthur Lira, who quickly managed to approve the urgent request for Bill 1904,^35^ known as the “Child Pregnancy Bill” or the “Rape Bill”, which proposed to criminalise abortion after 22 weeks, making it a crime of murder and equalising the sentence.

The bill was both a response to the STF's move to block the CFM resolution and a political test for President Lula. Sóstenes Cavalcanti, author of PL 1904, said: “The President sent a letter to evangelicals during the election campaign saying he was against abortion. We want to see if he will veto it. Let's put Lula to the test”.^36^ In other words, the practice of making abortion a political weapon has been consolidated in Brazil.

The second came with Constitutional Amendment Bill 29/2024,^37^ which proposes to change the Federal Constitution to consider fertilisation as the “starting point of human life”. Signed by more than 180 parliamentarians from the liberal right and the far right, this bill is awaiting a vote and, if approved, will not need the validation of the Chief Executive.^38^

But the attacks of the last decade have been countered by the active struggle of progressive deputies and senators in the National Congress, by the judiciary, by some health professionals and also by feminist movements that have taken to the streets, the press and social networks in defence of life and dignity.^39,40^

An example of this was the shelving of Bill 1904, following a significant mobilisation by the feminist movement that culminated in the popular protests of the “A child is not a mother” campaign.^41^ Held in July 2024, these protests took to the streets, drawing public attention to the impact of the bill on the lives of children and adolescents who are victims of sexual violence. The strength and response of the movement allowed for a political articulation that took place not only in the streets, but also in the media and social networks, with the support of Brazilian actresses, journalists and public figures. As a result, the bill was rejected by 90% of the population. In an open vote in the Chamber of Deputies, dozens of deputies withdrew their names from the bill, claiming to be unaware of its content. The mobilisation of the population has undoubtedly prevented a step backwards in the scenario of legal abortion, although no significant progress has been made.^42^

Other examples of resistance that have been articulated are strategic litigation actions to take emblematic cases of human rights violations to international courts^41^ and the expansion of communication strategies for social decriminalisation around the issue.^43^ There are also harm reduction actions^44,45^ and constant pressure to guarantee access to legal abortion services.^46^

Another important highlight should be the voting process at the federal court on the Argumentation of Noncompliance of Fundamental Precept (ADPF) 442, a measure proposed by the Socialism and Liberty Party (PSOL) and the Anis Bioethics Institute, which aims to decriminalise abortion up to the 12th week of pregnancy and which had its first public consultation in August 2018. The first round of the ADPF trial, which took place on the eve of the presidential election that elected Bolsonaro, was considered a battleground, with the STF listening to 50 organisations in a public hearing as *amicus curie*.^47^

In 2023, Judge Rosa Weber, in the final step of the ADPF 442 case, registered her vote in favour of decriminalisation, declaring her support for the defence of human rights and social and reproductive justice. No date has been set for further voting.

The impact of abortion-related gender discrimination in health services on everyday life is evident in many ways. At the end of 2023, one of the country's main legal abortion services had its activities suspended, allegedly due to “lack of demand” and irregularities in procedures.^48^ Health professionals are subject to judicial and political persecution^49,50^ and face criminal homicide charges in the CFM courts for performing abortions beyond 22 weeks^51^ even when within the law. Attempts at criminalisation also target professionals who are committed to the best scientific evidence and WHO recommendations, such as those against the implementation of a legal abortion service via telemedicine.^52^ Hospitals are refusing to perform abortions, postponing procedures until they become increasingly difficult to perform^53^ and pressuring pregnant individuals to assume parenthood or give the child up for adoption.^54,55^

It is difficult to accurately calculate the magnitude of the abortion problem in the country, which entails obtaining information on both the number of people whose right to legal abortion is violated and those who, faced with unforeseen pregnancies, are forced to resort to clandestine practices. This difficulty stems from a “deliberate blindness”, known as strategic ignorance,^26^ which leads the State to ignore both the extent and consequences of non-compliance with legislation and the high prevalence of clandestine practices generated by restrictive laws. This strategic ignorance upholds the fiction that abortion is an “exception”, perpetuates the status quo, and hides the fact that the State is complicit in the social and health risks imposed by restrictive laws which are not even implemented for all to who they apply.^26^

The growing academic interest in the subject^56,57^ serves a dual purpose: to deepen understanding of the context and subjects involved, and to fill the gap left by official data – both seen as scientific forms of resistance that contribute to the public debate. One of them shows the consequences of the huge gap between the law and reality: between 2011 and 2021, almost 130,000 girls aged between 10 and 14 years had children.^58^ According to Brazilian law, all of them could have been entitled to the procedure.

Meanwhile, another 101 bills are being processed in Congress that could, among other things, restrict, threaten or even invalidate legal abortion in Brazil, as well as increase the penalties for illegal abortions.^38^ The resistance forces, led by different actors, will be crucial to face these attacks, but the struggle is unceasing, urgent and challenging.

## Provenance statement

This article was not commissioned and went through external peer review.
